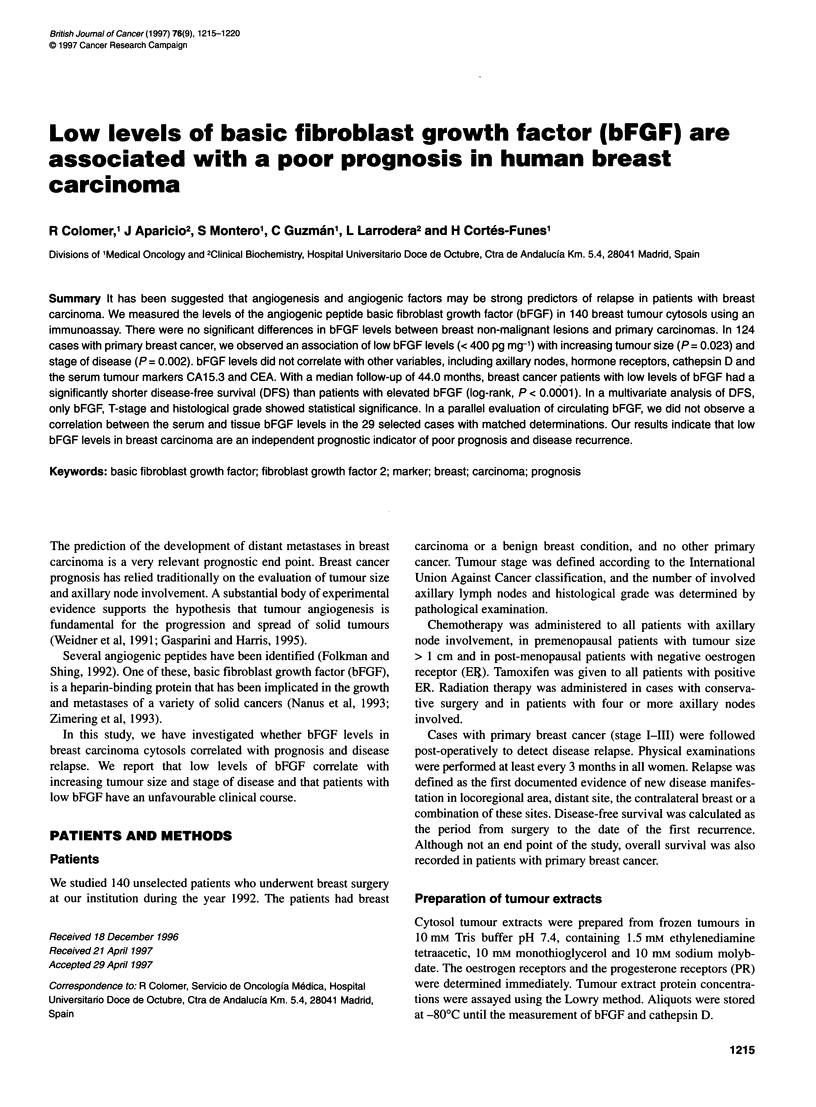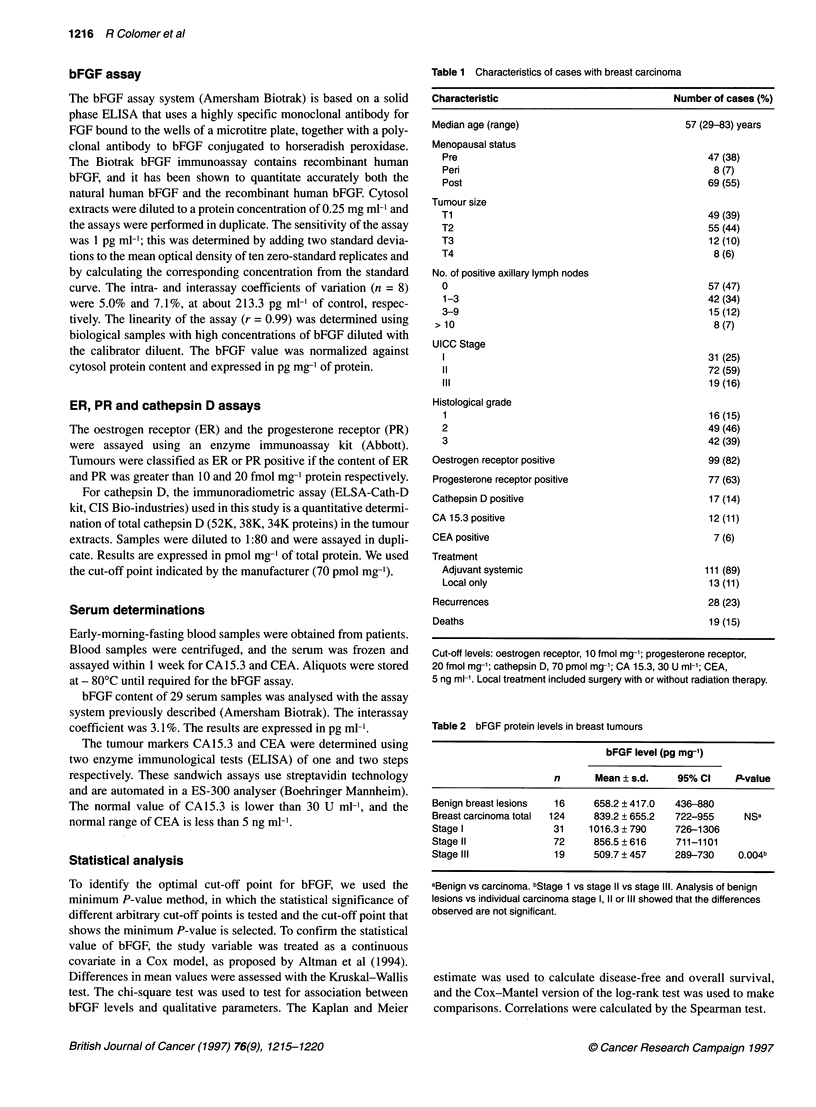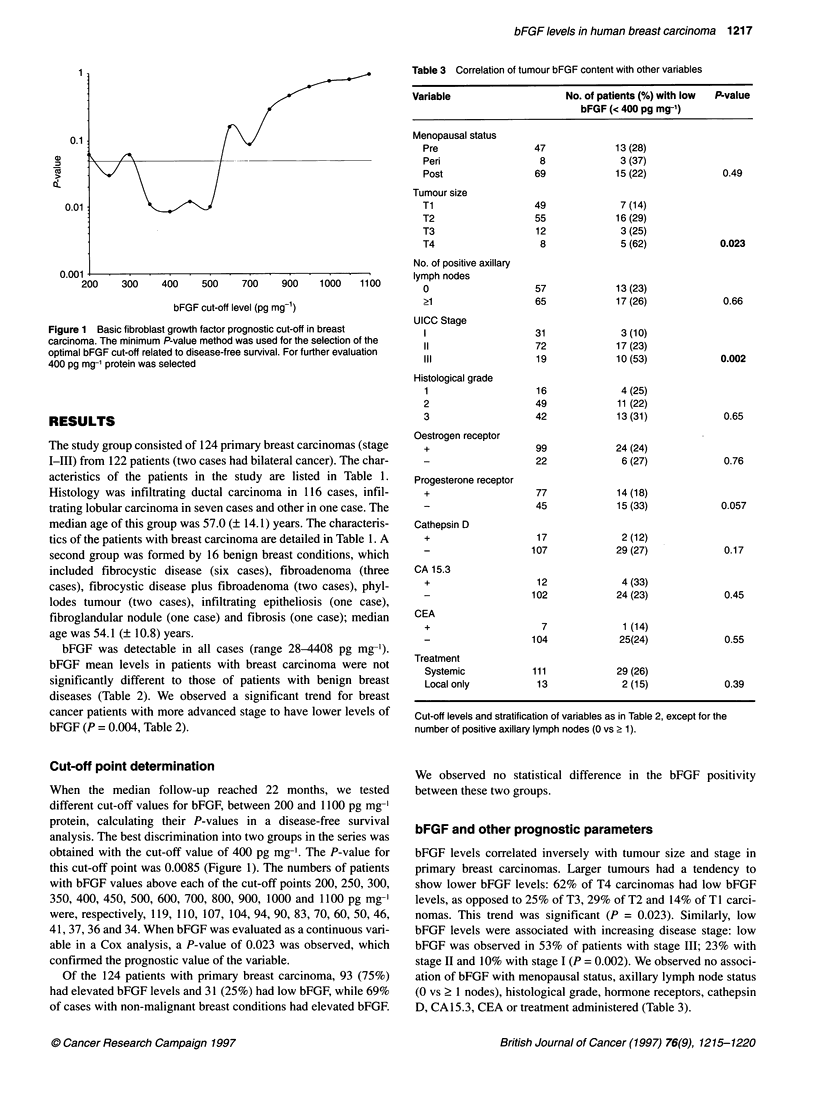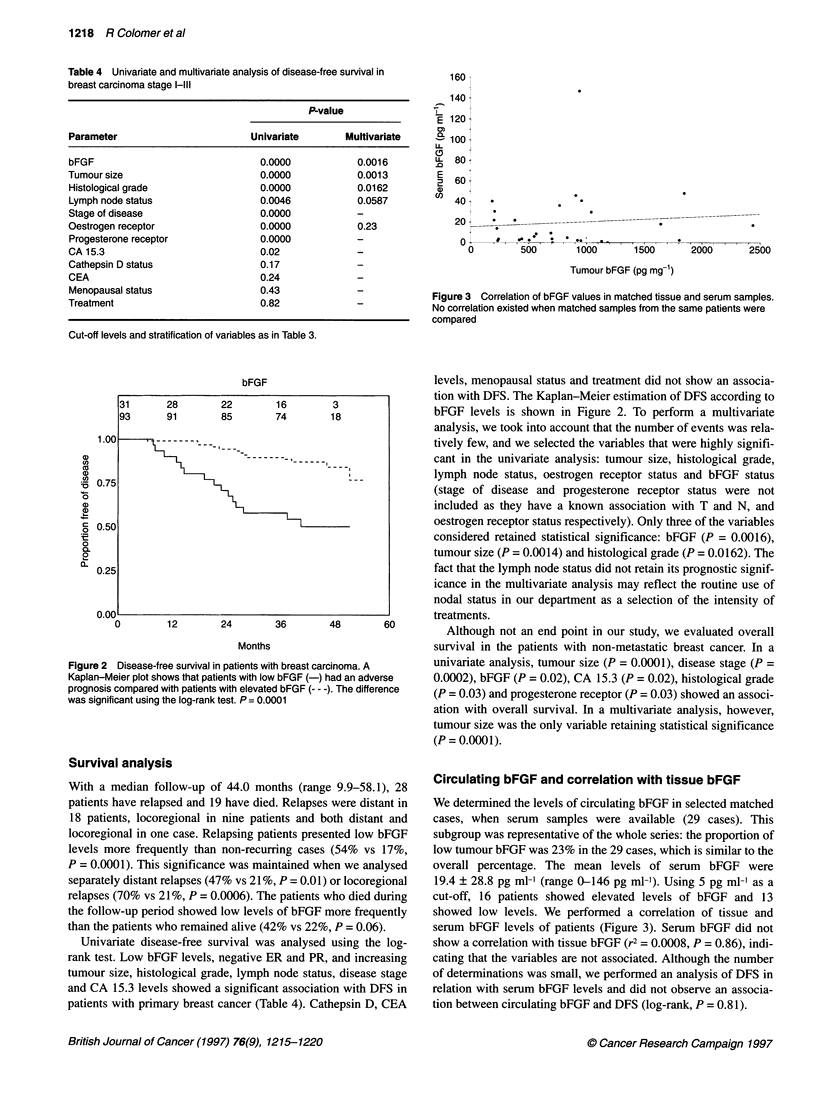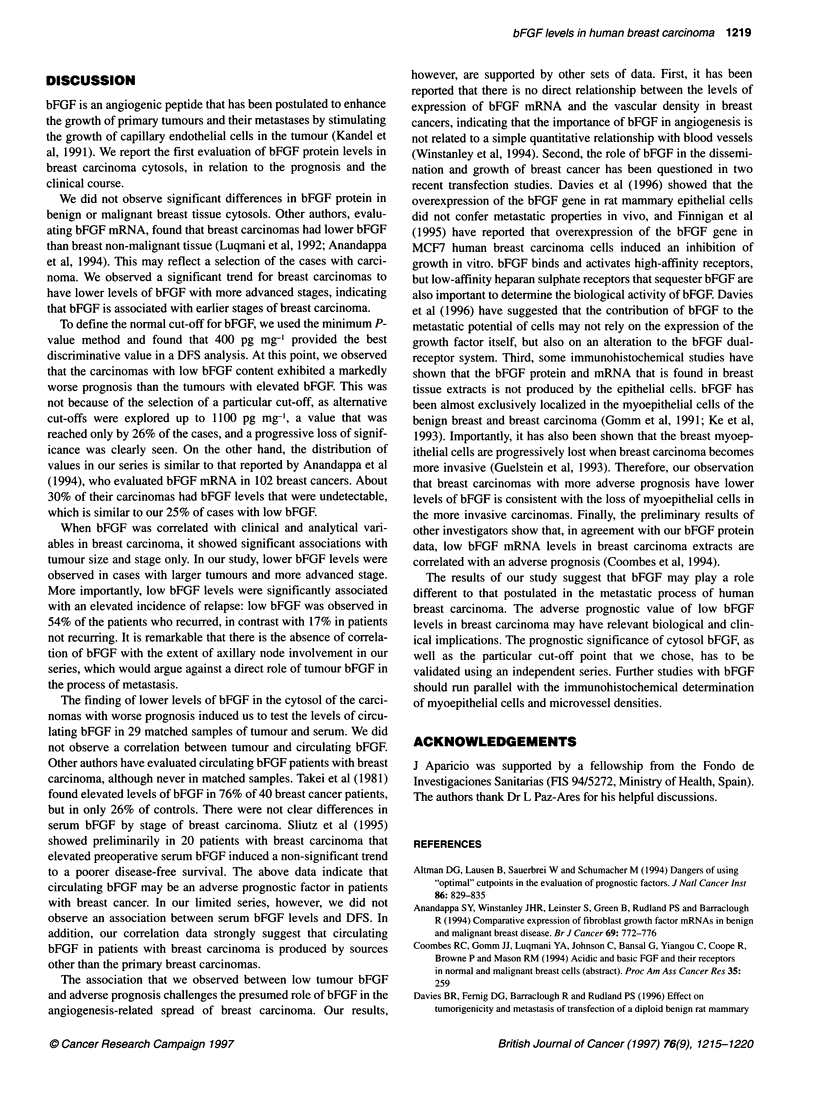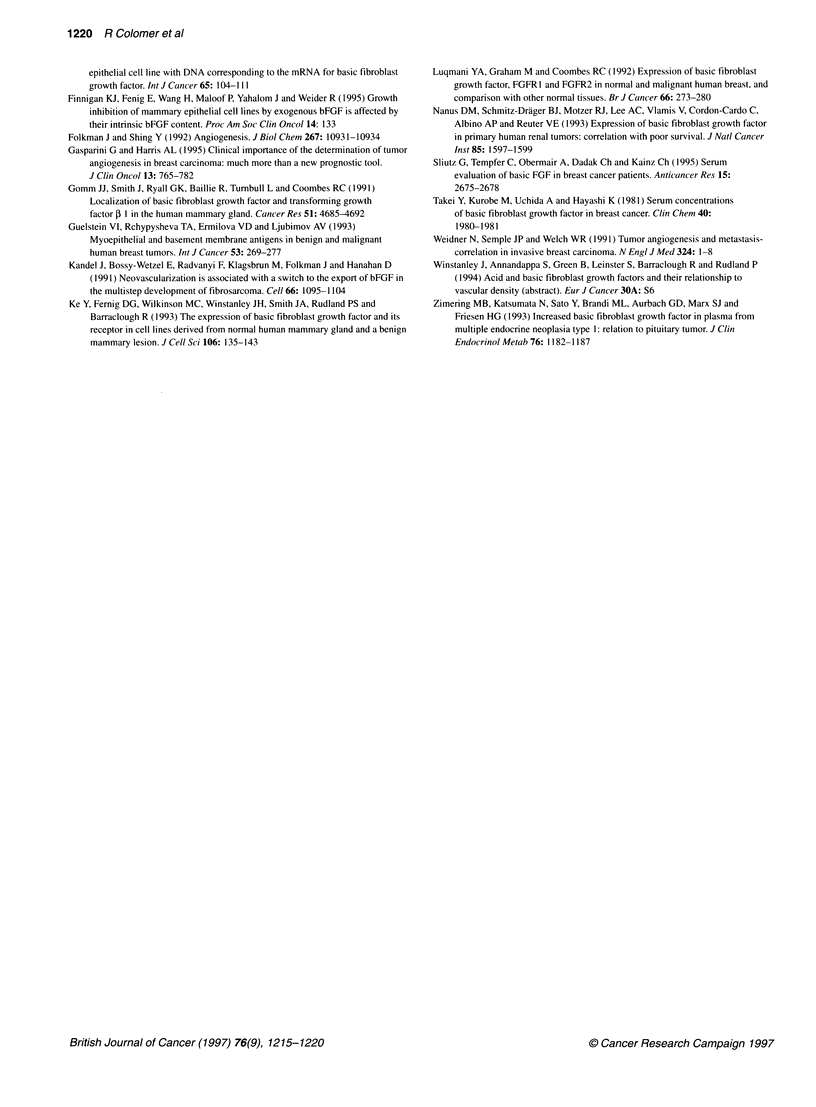# Low levels of basic fibroblast growth factor (bFGF) are associated with a poor prognosis in human breast carcinoma.

**DOI:** 10.1038/bjc.1997.536

**Published:** 1997

**Authors:** R. Colomer, J. Aparicio, S. Montero, C. GuzmÃ¡n, L. Larrodera, H. CortÃ©s-Funes

**Affiliations:** Division of Medical Oncology, Hospital Universitario Doce de Octubre, Madrid, Spain.

## Abstract

It has been suggested that angiogenesis and angiogenic factors may be strong predictors of relapse in patients with breast carcinoma. We measured the levels of the angiogenic peptide basic fibroblast growth factor (bFGF) in 140 breast tumour cytosols using an immunoassay. There were no significant differences in bFGF levels between breast non-malignant lesions and primary carcinomas. In 124 cases with primary breast cancer, we observed an association of low bFGF levels (< 400 pg mg[-1]) with increasing tumour size (P = 0.023) and stage of disease (P = 0.002). bFGF levels did not correlate with other variables, including axillary nodes, hormone receptors, cathepsin D and the serum tumour markers CA15.3 and CEA. With a median follow-up of 44.0 months, breast cancer patients with low levels of bFGF had a significantly shorter disease-free survival (DFS) than patients with elevated bFGF (log-rank, P < 0.0001). In a multivariate analysis of DFS, only bFGF, T-stage and histological grade showed statistical significance. In a parallel evaluation of circulating bFGF, we did not observe a correlation between the serum and tissue bFGF levels in the 29 selected cases with matched determinations. Our results indicate that low bFGF levels in breast carcinoma are an independent prognostic indicator of poor prognosis and disease recurrence.


*British Journal of Cancer* (1997) **76**(9), 1215–1220
© 1997 Cancer Research Campaign

### Low levels of basic fibroblast growth factor (bFGF) are associated with a poor prognosis in human breast carcinoma

**R Colomer,[1] J Aparicio[2], S Montero[1], C Guzmán[1], L Larrodera[2] and H Cortés-Funes[1]**

Divisions of [1]Medical Oncology and [2]Clinical Biochemistry, Hospital Universitario Doce de Octubre, Ctra de Andalucía Km. 5.4, 28041 Madrid, Spain

**Summary** It has been suggested that angiogenesis and angiogenic factors may be strong predictors of relapse in patients with breast carcinoma. We measured the levels of the angiogenic peptide basic fibroblast growth factor (bFGF) in 140 breast tumour cytosols using an immunoassay. There were no significant differences in bFGF levels between breast non-malignant lesions and primary carcinomas. In 124 cases with primary breast cancer, we observed an association of low bFGF levels (< 400 pg $mg^{-1}$) with increasing tumour size ($P = 0.023$) and stage of disease ($P = 0.002$). bFGF levels did not correlate with other variables, including axillary nodes, hormone receptors, cathepsin D and the serum tumour markers CA15.3 and CEA. With a median follow-up of 44.0 months, breast cancer patients with low levels of bFGF had a significantly shorter disease-free survival (DFS) than patients with elevated bFGF (log-rank, $P < 0.0001$). In a multivariate analysis of DFS, only bFGF, T-stage and histological grade showed statistical significance. In a parallel evaluation of circulating bFGF, we did not observe a correlation between the serum and tissue bFGF levels in the 29 selected cases with matched determinations. Our results indicate that low bFGF levels in breast carcinoma are an independent prognostic indicator of poor prognosis and disease recurrence.

**Keywords:** basic fibroblast growth factor; fibroblast growth factor 2; marker; breast; carcinoma; prognosis

The prediction of the development of distant metastases in breast carcinoma is a very relevant prognostic end point. Breast cancer prognosis has relied traditionally on the evaluation of tumour size and axillary node involvement. A substantial body of experimental evidence supports the hypothesis that tumour angiogenesis is fundamental for the progression and spread of solid tumours (Weidner et al, 1991; Gasparini and Harris, 1995).

Several angiogenic peptides have been identified (Folkman and Shing, 1992). One of these, basic fibroblast growth factor (bFGF), is a heparin-binding protein that has been implicated in the growth and metastases of a variety of solid cancers (Nanus et al, 1993; Zimering et al, 1993).

In this study, we have investigated whether bFGF levels in breast carcinoma cytosols correlated with prognosis and disease relapse. We report that low levels of bFGF correlate with increasing tumour size and stage of disease and that patients with low bFGF have an unfavourable clinical course.

### PATIENTS AND METHODS

### Patients

We studied 140 unselected patients who underwent breast surgery at our institution during the year 1992. The patients had breast carcinoma or a benign breast condition, and no other primary cancer. Tumour stage was defined according to the International Union Against Cancer classification, and the number of involved axillary lymph nodes and histological grade was determined by pathological examination.

Chemotherapy was administered to all patients with axillary node involvement, in premenopausal patients with tumour size > 1 cm and in post-menopausal patients with negative oestrogen receptor (ER). Tamoxifen was given to all patients with positive ER. Radiation therapy was administered in cases with conservative surgery and in patients with four or more axillary nodes involved.

Cases with primary breast cancer (stage I–III) were followed post-operatively to detect disease relapse. Physical examinations were performed at least every 3 months in all women. Relapse was defined as the first documented evidence of new disease manifestation in locoregional area, distant site, the contralateral breast or a combination of these sites. Disease-free survival was calculated as the period from surgery to the date of the first recurrence. Although not an end point of the study, overall survival was also recorded in patients with primary breast cancer.

*Received 18 December 1996*
*Received 21 April 1997*
*Accepted 29 April 1997*

*Correspondence to:* R Colomer, Servicio de Oncología Médica, Hospital Universitario Doce de Octubre, Ctra de Andalucía Km. 5.4, 28041 Madrid, Spain

### Preparation of tumour extracts

Cytosol tumour extracts were prepared from frozen tumours in 10 mM Tris buffer pH 7.4, containing 1.5 mM ethylenediamine tetraacetic, 10 mM monothioglycerol and 10 mM sodium molybdate. The oestrogen receptors and the progesterone receptors (PR) were determined immediately. Tumour extract protein concentrations were assayed using the Lowry method. Aliquots were stored at –80°C until the measurement of bFGF and cathepsin D.

### bFGF assay

The bFGF assay system (Amersham Biotrak) is based on a solid phase ELISA that uses a highly specific monoclonal antibody for FGF bound to the wells of a microtitre plate, together with a polyclonal antibody to bFGF conjugated to horseradish peroxidase. The Biotrak bFGF immunoassay contains recombinant human bFGF, and it has been shown to quantitate accurately both the natural human bFGF and the recombinant human bFGF. Cytosol extracts were diluted to a protein concentration of 0.25 mg $ml^{-1}$ and the assays were performed in duplicate. The sensitivity of the assay was 1 pg $ml^{-1}$; this was determined by adding two standard deviations to the mean optical density of ten zero-standard replicates and by calculating the corresponding concentration from the standard curve. The intra- and interassay coefficients of variation ($n$ = 8) were 5.0% and 7.1%, at about 213.3 pg $ml^{-1}$ of control, respectively. The linearity of the assay ($r$ = 0.99) was determined using biological samples with high concentrations of bFGF diluted with the calibrator diluent. The bFGF value was normalized against cytosol protein content and expressed in pg $mg^{-1}$ of protein.

### ER, PR and cathepsin D assays

The oestrogen receptor (ER) and the progesterone receptor (PR) were assayed using an enzyme immunoassay kit (Abbott). Tumours were classified as ER or PR positive if the content of ER and PR was greater than 10 and 20 fmol $mg^{-1}$ protein respectively.

For cathepsin D, the immunoradiometric assay (ELSA-Cath-D kit, CIS Bio-industries) used in this study is a quantitative determination of total cathepsin D (52K, 38K, 34K proteins) in the tumour extracts. Samples were diluted to 1:80 and were assayed in duplicate. Results are expressed in pmol $mg^{-1}$ of total protein. We used the cut-off point indicated by the manufacturer (70 pmol $mg^{-1}$).

### Serum determinations

Early-morning-fasting blood samples were obtained from patients. Blood samples were centrifuged, and the serum was frozen and assayed within 1 week for CA15.3 and CEA. Aliquots were stored at – 80°C until required for the bFGF assay.

bFGF content of 29 serum samples was analysed with the assay system previously described (Amersham Biotrak). The interassay coefficient was 3.1%. The results are expressed in pg $ml^{-1}$.

The tumour markers CA15.3 and CEA were determined using two enzyme immunological tests (ELISA) of one and two steps respectively. These sandwich assays use streptavidin technology and are automated in a ES-300 analyser (Boehringer Mannheim). The normal value of CA15.3 is lower than 30 U $ml^{-1}$, and the normal range of CEA is less than 5 ng $ml^{-1}$.

### Statistical analysis

To identify the optimal cut-off point for bFGF, we used the minimum $P$-value method, in which the statistical significance of different arbitrary cut-off points is tested and the cut-off point that shows the minimum $P$-value is selected. To confirm the statistical value of bFGF, the study variable was treated as a continuous covariate in a Cox model, as proposed by Altman et al (1994). Differences in mean values were assessed with the Kruskal–Wallis test. The chi-square test was used to test for association between bFGF levels and qualitative parameters. The Kaplan and Meier estimate was used to calculate disease-free and overall survival, and the Cox–Mantel version of the log-rank test was used to make comparisons. Correlations were calculated by the Spearman test.

**Table 1** Characteristics of cases with breast carcinoma

| Characteristic | Number of cases (%) |
|---|---|
| Median age (range) | 57 (29–83) years |
| Menopausal status | |
| Pre | 47 (38) |
| Peri | 8 (7) |
| Post | 69 (55) |
| Tumour size | |
| T1 | 49 (39) |
| T2 | 55 (44) |
| T3 | 12 (10) |
| T4 | 8 (6) |
| No. of positive axillary lymph nodes | |
| 0 | 57 (47) |
| 1–3 | 42 (34) |
| 3–9 | 15 (12) |
| > 10 | 8 (7) |
| UICC Stage | |
| I | 31 (25) |
| II | 72 (59) |
| III | 19 (16) |
| Histological grade | |
| 1 | 16 (15) |
| 2 | 49 (46) |
| 3 | 42 (39) |
| Oestrogen receptor positive | 99 (82) |
| Progesterone receptor positive | 77 (63) |
| Cathepsin D positive | 17 (14) |
| CA 15.3 positive | 12 (11) |
| CEA positive | 7 (6) |
| Treatment | |
| Adjuvant systemic | 111 (89) |
| Local only | 13 (11) |
| Recurrences | 28 (23) |
| Deaths | 19 (15) |

Cut-off levels: oestrogen receptor, 10 fmol $mg^{-1}$; progesterone receptor, 20 fmol $mg^{-1}$; cathepsin D, 70 pmol $mg^{-1}$; CA 15.3, 30 U $ml^{-1}$; CEA, 5 ng $ml^{-1}$. Local treatment included surgery with or without radiation therapy.

**Table 2** bFGF protein levels in breast tumours

| | | bFGF level (pg $mg^{-1}$) | | |
|---|---|---|---|---|
| | *n* | Mean ± s.d. | 95% CI | *P*-value |
| Benign breast lesions | 16 | 658.2 ± 417.0 | 436–880 | |
| Breast carcinoma total | 124 | 839.2 ± 655.2 | 722–955 | NS[a] |
| Stage I | 31 | 1016.3 ± 790 | 726–1306 | |
| Stage II | 72 | 856.5 ± 616 | 711–1101 | |
| Stage III | 19 | 509.7 ± 457 | 289–730 | 0.004[b] |

[a]Benign vs carcinoma. [b]Stage 1 vs stage II vs stage III. Analysis of benign lesions vs individual carcinoma stage I, II or III showed that the differences observed are not significant.

*© Cancer Research Campaign 1997*

**Figure 1** Basic fibroblast growth factor prognostic cut-off in breast carcinoma. The minimum *P*-value method was used for the selection of the optimal bFGF cut-off related to disease-free survival. For further evaluation 400 pg mg$^{-1}$ protein was selected

### RESULTS

The study group consisted of 124 primary breast carcinomas (stage I–III) from 122 patients (two cases had bilateral cancer). The characteristics of the patients in the study are listed in Table 1. Histology was infiltrating ductal carcinoma in 116 cases, infiltrating lobular carcinoma in seven cases and other in one case. The median age of this group was 57.0 (± 14.1) years. The characteristics of the patients with breast carcinoma are detailed in Table 1. A second group was formed by 16 benign breast conditions, which included fibrocystic disease (six cases), fibroadenoma (three cases), fibrocystic disease plus fibroadenoma (two cases), phyllodes tumour (two cases), infiltrating epitheliosis (one case), fibroglandular nodule (one case) and fibrosis (one case); median age was 54.1 (± 10.8) years.

bFGF was detectable in all cases (range 28–4408 pg mg$^{-1}$). bFGF mean levels in patients with breast carcinoma were not significantly different to those of patients with benign breast diseases (Table 2). We observed a significant trend for breast cancer patients with more advanced stage to have lower levels of bFGF ($P = 0.004$, Table 2).

### Cut-off point determination

When the median follow-up reached 22 months, we tested different cut-off values for bFGF, between 200 and 1100 pg mg$^{-1}$ protein, calculating their *P*-values in a disease-free survival analysis. The best discrimination into two groups in the series was obtained with the cut-off value of 400 pg mg$^{-1}$. The *P*-value for this cut-off point was 0.0085 (Figure 1). The numbers of patients with bFGF values above each of the cut-off points 200, 250, 300, 350, 400, 450, 500, 600, 700, 800, 900, 1000 and 1100 pg mg$^{-1}$ were, respectively, 119, 110, 107, 104, 94, 90, 83, 70, 60, 50, 46, 41, 37, 36 and 34. When bFGF was evaluated as a continuous variable in a Cox analysis, a *P*-value of 0.023 was observed, which confirmed the prognostic value of the variable.

Of the 124 patients with primary breast carcinoma, 93 (75%) had elevated bFGF levels and 31 (25%) had low bFGF, while 69% of cases with non-malignant breast conditions had elevated bFGF.

**Table 3** Correlation of tumour bFGF content with other variables

| Variable | | No. of patients (%) with low bFGF (< 400 pg mg$^{-1}$) | *P*-value |
|---|---|---|---|
| Menopausal status | | | |
| Pre | 47 | 13 (28) | |
| Peri | 8 | 3 (37) | |
| Post | 69 | 15 (22) | 0.49 |
| Tumour size | | | |
| T1 | 49 | 7 (14) | |
| T2 | 55 | 16 (29) | |
| T3 | 12 | 3 (25) | |
| T4 | 8 | 5 (62) | **0.023** |
| No. of positive axillary lymph nodes | | | |
| 0 | 57 | 13 (23) | |
| ≥1 | 65 | 17 (26) | 0.66 |
| UICC Stage | | | |
| I | 31 | 3 (10) | |
| II | 72 | 17 (23) | |
| III | 19 | 10 (53) | **0.002** |
| Histological grade | | | |
| 1 | 16 | 4 (25) | |
| 2 | 49 | 11 (22) | |
| 3 | 42 | 13 (31) | 0.65 |
| Oestrogen receptor | | | |
| + | 99 | 24 (24) | |
| – | 22 | 6 (27) | 0.76 |
| Progesterone receptor | | | |
| + | 77 | 14 (18) | |
| – | 45 | 15 (33) | 0.057 |
| Cathepsin D | | | |
| + | 17 | 2 (12) | |
| – | 107 | 29 (27) | 0.17 |
| CA 15.3 | | | |
| + | 12 | 4 (33) | |
| – | 102 | 24 (23) | 0.45 |
| CEA | | | |
| + | 7 | 1 (14) | |
| – | 104 | 25(24) | 0.55 |
| Treatment | | | |
| Systemic | 111 | 29 (26) | |
| Local only | 13 | 2 (15) | 0.39 |

Cut-off levels and stratification of variables as in Table 2, except for the number of positive axillary lymph nodes (0 vs ≥ 1).

We observed no statistical difference in the bFGF positivity between these two groups.

### bFGF and other prognostic parameters bFGF levels correlated inversely with tumour size and stage in primary breast carcinomas. Larger tumours had a tendency to show lower bFGF levels: 62% of T4 carcinomas had low bFGF levels, as opposed to 25% of T3, 29% of T2 and 14% of T1 carcinomas. This trend was significant ($P = 0.023$). Similarly, low bFGF levels were associated with increasing disease stage: low bFGF was observed in 53% of patients with stage III; 23% with stage II and 10% with stage I ($P = 0.002$). We observed no association of bFGF with menopausal status, axillary lymph node status (0 vs ≥ 1 nodes), histological grade, hormone receptors, cathepsin D, CA15.3, CEA or treatment administered (Table 3).

© Cancer Research Campaign 1997

**Table 4** Univariate and multivariate analysis of disease-free survival in breast carcinoma stage I–III

| Parameter | P-value Univariate | P-value Multivariate |
|---|---|---|
| bFGF | 0.0000 | 0.0016 |
| Tumour size | 0.0000 | 0.0013 |
| Histological grade | 0.0000 | 0.0162 |
| Lymph node status | 0.0046 | 0.0587 |
| Stage of disease | 0.0000 | – |
| Oestrogen receptor | 0.0000 | 0.23 |
| Progesterone receptor | 0.0000 | – |
| CA 15.3 | 0.02 | – |
| Cathepsin D status | 0.17 | – |
| CEA | 0.24 | – |
| Menopausal status | 0.43 | – |
| Treatment | 0.82 | – |

Cut-off levels and stratification of variables as in Table 3.

**Figure 3** Correlation of bFGF values in matched tissue and serum samples. No correlation existed when matched samples from the same patients were compared

**Figure 2** Disease-free survival in patients with breast carcinoma. A Kaplan–Meier plot shows that patients with low bFGF (—) had an adverse prognosis compared with patients with elevated bFGF (- - -). The difference was significant using the log-rank test. $P = 0.0001$

### Survival analysis

With a median follow-up of 44.0 months (range 9.9–58.1), 28 patients have relapsed and 19 have died. Relapses were distant in 18 patients, locoregional in nine patients and both distant and locoregional in one case. Relapsing patients presented low bFGF levels more frequently than non-recurring cases (54% vs 17%, $P = 0.0001$). This significance was maintained when we analysed separately distant relapses (47% vs 21%, $P = 0.01$) or locoregional relapses (70% vs 21%, $P = 0.0006$). The patients who died during the follow-up period showed low levels of bFGF more frequently than the patients who remained alive (42% vs 22%, $P = 0.06$).

Univariate disease-free survival was analysed using the log-rank test. Low bFGF levels, negative ER and PR, and increasing tumour size, histological grade, lymph node status, disease stage and CA 15.3 levels showed a significant association with DFS in patients with primary breast cancer (Table 4). Cathepsin D, CEA levels, menopausal status and treatment did not show an association with DFS. The Kaplan–Meier estimation of DFS according to bFGF levels is shown in Figure 2. To perform a multivariate analysis, we took into account that the number of events was relatively few, and we selected the variables that were highly significant in the univariate analysis: tumour size, histological grade, lymph node status, oestrogen receptor status and bFGF status (stage of disease and progesterone receptor status were not included as they have a known association with T and N, and oestrogen receptor status respectively). Only three of the variables considered retained statistical significance: bFGF ($P = 0.0016$), tumour size ($P = 0.0014$) and histological grade ($P = 0.0162$). The fact that the lymph node status did not retain its prognostic significance in the multivariate analysis may reflect the routine use of nodal status in our department as a selection of the intensity of treatments.

Although not an end point in our study, we evaluated overall survival in the patients with non-metastatic breast cancer. In a univariate analysis, tumour size ($P = 0.0001$), disease stage ($P = 0.0002$), bFGF ($P = 0.02$), CA 15.3 ($P = 0.02$), histological grade ($P = 0.03$) and progesterone receptor ($P = 0.03$) showed an association with overall survival. In a multivariate analysis, however, tumour size was the only variable retaining statistical significance ($P = 0.0001$).

### Circulating bFGF and correlation with tissue bFGF

We determined the levels of circulating bFGF in selected matched cases, when serum samples were available (29 cases). This subgroup was representative of the whole series: the proportion of low tumour bFGF was 23% in the 29 cases, which is similar to the overall percentage. The mean levels of serum bFGF were 19.4 ± 28.8 pg $ml^{-1}$ (range 0–146 pg $ml^{-1}$). Using 5 pg $ml^{-1}$ as a cut-off, 16 patients showed elevated levels of bFGF and 13 showed low levels. We performed a correlation of tissue and serum bFGF levels of patients (Figure 3). Serum bFGF did not show a correlation with tissue bFGF ($r^2 = 0.0008$, $P = 0.86$), indicating that the variables are not associated. Although the number of determinations was small, we performed an analysis of DFS in relation with serum bFGF levels and did not observe an association between circulating bFGF and DFS (log-rank, $P = 0.81$).

© Cancer Research Campaign 1997

### DISCUSSION

bFGF is an angiogenic peptide that has been postulated to enhance the growth of primary tumours and their metastases by stimulating the growth of capillary endothelial cells in the tumour (Kandel et al, 1991). We report the first evaluation of bFGF protein levels in breast carcinoma cytosols, in relation to the prognosis and the clinical course.

We did not observe significant differences in bFGF protein in benign or malignant breast tissue cytosols. Other authors, evaluating bFGF mRNA, found that breast carcinomas had lower bFGF than breast non-malignant tissue (Luqmani et al, 1992; Anandappa et al, 1994). This may reflect a selection of the cases with carcinoma. We observed a significant trend for breast carcinomas to have lower levels of bFGF with more advanced stages, indicating that bFGF is associated with earlier stages of breast carcinoma.

To define the normal cut-off for bFGF, we used the minimum *P*-value method and found that 400 pg $mg^{-1}$ provided the best discriminative value in a DFS analysis. At this point, we observed that the carcinomas with low bFGF content exhibited a markedly worse prognosis than the tumours with elevated bFGF. This was not because of the selection of a particular cut-off, as alternative cut-offs were explored up to 1100 pg $mg^{-1}$, a value that was reached only by 26% of the cases, and a progressive loss of significance was clearly seen. On the other hand, the distribution of values in our series is similar to that reported by Anandappa et al (1994), who evaluated bFGF mRNA in 102 breast cancers. About 30% of their carcinomas had bFGF levels that were undetectable, which is similar to our 25% of cases with low bFGF.

When bFGF was correlated with clinical and analytical variables in breast carcinoma, it showed significant associations with tumour size and stage only. In our study, lower bFGF levels were observed in cases with larger tumours and more advanced stage. More importantly, low bFGF levels were significantly associated with an elevated incidence of relapse: low bFGF was observed in 54% of the patients who recurred, in contrast with 17% in patients not recurring. It is remarkable that there is the absence of correlation of bFGF with the extent of axillary node involvement in our series, which would argue against a direct role of tumour bFGF in the process of metastasis.

The finding of lower levels of bFGF in the cytosol of the carcinomas with worse prognosis induced us to test the levels of circulating bFGF in 29 matched samples of tumour and serum. We did not observe a correlation between tumour and circulating bFGF. Other authors have evaluated circulating bFGF patients with breast carcinoma, although never in matched samples. Takei et al (1981) found elevated levels of bFGF in 76% of 40 breast cancer patients, but in only 26% of controls. There were not clear differences in serum bFGF by stage of breast carcinoma. Sliutz et al (1995) showed preliminarily in 20 patients with breast carcinoma that elevated preoperative serum bFGF induced a non-significant trend to a poorer disease-free survival. The above data indicate that circulating bFGF may be an adverse prognostic factor in patients with breast cancer. In our limited series, however, we did not observe an association between serum bFGF levels and DFS. In addition, our correlation data strongly suggest that circulating bFGF in patients with breast carcinoma is produced by sources other than the primary breast carcinomas.

The association that we observed between low tumour bFGF and adverse prognosis challenges the presumed role of bFGF in the angiogenesis-related spread of breast carcinoma. Our results, however, are supported by other sets of data. First, it has been reported that there is no direct relationship between the levels of expression of bFGF mRNA and the vascular density in breast cancers, indicating that the importance of bFGF in angiogenesis is not related to a simple quantitative relationship with blood vessels (Winstanley et al, 1994). Second, the role of bFGF in the dissemination and growth of breast cancer has been questioned in two recent transfection studies. Davies et al (1996) showed that the overexpression of the bFGF gene in rat mammary epithelial cells did not confer metastatic properties in vivo, and Finnigan et al (1995) have reported that overexpression of the bFGF gene in MCF7 human breast carcinoma cells induced an inhibition of growth in vitro. bFGF binds and activates high-affinity receptors, but low-affinity heparan sulphate receptors that sequester bFGF are also important to determine the biological activity of bFGF. Davies et al (1996) have suggested that the contribution of bFGF to the metastatic potential of cells may not rely on the expression of the growth factor itself, but also on an alteration to the bFGF dual-receptor system. Third, some immunohistochemical studies have shown that the bFGF protein and mRNA that is found in breast tissue extracts is not produced by the epithelial cells. bFGF has been almost exclusively localized in the myoepithelial cells of the benign breast and breast carcinoma (Gomm et al, 1991; Ke et al, 1993). Importantly, it has also been shown that the breast myoepithelial cells are progressively lost when breast carcinoma becomes more invasive (Guelstein et al, 1993). Therefore, our observation that breast carcinomas with more adverse prognosis have lower levels of bFGF is consistent with the loss of myoepithelial cells in the more invasive carcinomas. Finally, the preliminary results of other investigators show that, in agreement with our bFGF protein data, low bFGF mRNA levels in breast carcinoma extracts are correlated with an adverse prognosis (Coombes et al, 1994).

The results of our study suggest that bFGF may play a role different to that postulated in the metastatic process of human breast carcinoma. The adverse prognostic value of low bFGF levels in breast carcinoma may have relevant biological and clinical implications. The prognostic significance of cytosol bFGF, as well as the particular cut-off point that we chose, has to be validated using an independent series. Further studies with bFGF should run parallel with the immunohistochemical determination of myoepithelial cells and microvessel densities.

### ACKNOWLEDGEMENTS

J Aparicio was supported by a fellowship from the Fondo de Investigaciones Sanitarias (FIS 94/5272, Ministry of Health, Spain). The authors thank Dr L Paz-Ares for his helpful discussions.

### REFERENCES

Altman DG, Lausen B, Sauerbrei W and Schumacher M (1994) Dangers of using "optimal" cutpoints in the evaluation of prognostic factors. *J Natl Cancer Inst* **86:** 829–835

Anandappa SY, Winstanley JHR, Leinster S, Green B, Rudland PS and Barraclough R (1994) Comparative expression of fibroblast growth factor mRNAs in benign and malignant breast disease. *Br J Cancer* **69:** 772–776

Coombes RC, Gomm JJ, Luqmani YA, Johnson C, Bansal G, Yiangou C, Coope R, Browne P and Mason RM (1994) Acidic and basic FGF and their receptors in normal and malignant breast cells (abstract). *Proc Am Ass Cancer Res* **35:** 259

Davies BR, Fernig DG, Barraclough R and Rudland PS (1996) Effect on tumorigenicity and metastasis of transfection of a diploid benign rat mammary

© Cancer Research Campaign 1997

epithelial cell line with DNA corresponding to the mRNA for basic fibroblast growth factor. *Int J Cancer* **65:** 104–111

Finnigan KJ, Fenig E, Wang H, Maloof P, Yahalom J and Weider R (1995) Growth inhibition of mammary epithelial cell lines by exogenous bFGF is affected by their intrinsic bFGF content. *Proc Am Soc Clin Oncol* **14**: 133

Folkman J and Shing Y (1992) Angiogenesis. *J Biol Chem* **267:** 10931–10934

Gasparini G and Harris AL (1995) Clinical importance of the determination of tumor angiogenesis in breast carcinoma: much more than a new prognostic tool. *J Clin Oncol* **13:** 765–782

Gomm JJ, Smith J, Ryall GK, Baillie R, Turnbull L and Coombes RC (1991) Localization of basic fibroblast growth factor and transforming growth factor β 1 in the human mammary gland. *Cancer Res* **51:** 4685–4692

Guelstein VI, Rchypysheva TA, Ermilova VD and Ljubimov AV (1993) Myoepithelial and basement membrane antigens in benign and malignant human breast tumors. *Int J Cancer* **53:** 269–277

Kandel J, Bossy-Wetzel E, Radvanyi F, Klagsbrun M, Folkman J and Hanahan D (1991) Neovascularization is associated with a switch to the export of bFGF in the multistep development of fibrosarcoma. *Cell* **66:** 1095–1104

Ke Y, Fernig DG, Wilkinson MC, Winstanley JH, Smith JA, Rudland PS and Barraclough R (1993) The expression of basic fibroblast growth factor and its receptor in cell lines derived from normal human mammary gland and a benign mammary lesion. *J Cell Sci* **106:** 135–143

Luqmani YA, Graham M and Coombes RC (1992) Expression of basic fibroblast growth factor, FGFR1 and FGFR2 in normal and malignant human breast, and comparison with other normal tissues. *Br J Cancer* **66:** 273–280

Nanus DM, Schmitz-Dräger BJ, Motzer RJ, Lee AC, Vlamis V, Cordon-Cardo C, Albino AP and Reuter VE (1993) Expression of basic fibroblast growth factor in primary human renal tumors: correlation with poor survival. *J Natl Cancer Inst* **85:** 1597–1599

Sliutz G, Tempfer C, Obermair A, Dadak Ch and Kainz Ch (1995) Serum evaluation of basic FGF in breast cancer patients. *Anticancer Res* **15:** 2675–2678

Takei Y, Kurobe M, Uchida A and Hayashi K (1981) Serum concentrations of basic fibroblast growth factor in breast cancer. *Clin Chem* **40:** 1980–1981

Weidner N, Semple JP and Welch WR (1991) Tumor angiogenesis and metastasis-correlation in invasive breast carcinoma. *N Engl J Med* **324:** 1–8

Winstanley J, Annandappa S, Green B, Leinster S, Barraclough R and Rudland P (1994) Acid and basic fibroblast growth factors and their relationship to vascular density (abstract). *Eur J Cancer* **30A:** S6

Zimering MB, Katsumata N, Sato Y, Brandi ML, Aurbach GD, Marx SJ and Friesen HG (1993) Increased basic fibroblast growth factor in plasma from multiple endocrine neoplasia type 1: relation to pituitary tumor. *J Clin Endocrinol Metab* **76:** 1182–1187

© Cancer Research Campaign 1997